# Assessment of Homologous Recombination System Gene Expression in Chemologically Induced Carcinogenesis In Vivo Models

**DOI:** 10.3390/cimb48030275

**Published:** 2026-03-04

**Authors:** Matvey M. Tsyganov, Danna Zh. Bulatova, Anastasia A. Fedorenko, Dmitry M. Loos, Pavel E. Nikiforov, Irina A. Tsydenova, Aigerim A. Bayanbayeva, Zhansaya Sharipkhanova, Sofia S. Timoshenko, Marina K. Ibragimova

**Affiliations:** 1 Cancer Research Institute, Tomsk National Research Medical Center of the Russian Academy of Sciences, St. Kooperativny 5, Tomsk 634009, Russia; 2 Biochemistry and Molecular Biology Division, Siberian State Medical University, 2 Moskovsky Trakt, Tomsk 634050, Russia; 3 Goldberg Research Institute of Pharmacology and Regenerative Medicine, 3 Lenin Avenue, Tomsk 634028, Russia

**Keywords:** chemo-induced carcinogenesis, homologous recombination genes, *Brca1*, expression, 3-methylcholanthrene, trichloroacetic acid, chemosensitivity

## Abstract

Understanding the molecular mechanisms of carcinogenesis, including disruptions in the homologous recombination system, is fundamental to understanding malignant transformation. Dysfunction of homologous recombination genes, such as *BRCA1* and *BRCA2*, contributes to genomic instability and the development of more aggressive tumor clones. The use of chemical carcinogens enables the modeling of tumor formation and the monitoring of changes in molecular genetic parameters. This approach is important for understanding how tumor cells adapt to genotoxic stress and for advancing the development of personalized cancer therapies. The objective of this study was to evaluate the expression of key homologous recombination system genes in a model of chemically induced carcinogenesis in mice. Materials and Methods: Male outbred ICR (CD-1) laboratory mice (*n* = 40) were used to study chemically induced carcinogenesis. The animals were divided into four groups: two control groups and two experimental groups, which received 3-methylcholanthrene (MC) or trichloroacetic acid (TCA). Tumor cells were identified by histological analysis of autopsy material using light microscopy after standard hematoxylin and eosin staining. RNA and DNA were extracted from cell suspensions using the RNeasy Plus Mini Kit and QIAamp DNA Mini Kit (Qiagen, Hilden, Germany), respectively. The expression levels of homologous recombination genes were assessed by RT-PCR and microarray analysis. Digital PCR was performed to assess chromosomal aberrations in the *Brca1* gene. Results: Tumor formations were identified in laboratory animals two months after 3-methylcholanthrene. Histological analysis revealed morphological changes in a pleomorphic cell tumor, forming diverse, multidirectional fascicular and swirling structures, as well as large solid foci composed of markedly polymorphic spindle-shaped and epithelioid cells. Analysis of copy number aberrations in the examined samples showed that the frequency of *Brca1* deletions was 60%, while 40% of animals had normal gene copy number. To further characterize the molecular changes, we assessed gene expression levels through expression microarray analysis. A total of 14 genes were hypoexpressed in the tumor compared to the normal tissue, with *p* < 0.05. A high level of differential expression was characteristic for *Rad50*, *Rad51*, *Brca1, Brca2*, and *Pold4*. Two genes, *Rad52* and *Bard1*, exhibited increased expression levels. It was shown that as the tumor mass increased, so did the frequency of homologous recombination genes with hypoexpression. Conclusions: Our findings confirm that MC and TCA influence tumor formation and reveal that suppression of homologous recombination genes may contribute to this process. In addition, it has been established that as tumors progress, the expression of DNA repair genes declines and aberrant gene states accumulate. These data emphasize the importance of studying the state of DNA repair genes for the development of more effective strategies for cancer diagnosis and therapy.

## 1. Introduction

Carcinogenesis is a complex, multi-step process in which normal cells accumulate genetic and epigenetic changes, leading to their malignant transformation. Studying the mechanisms underlying the origin and progression of solid tumors remains one of the most pressing challenges in modern biomedicine [1]. One of the key events underlying carcinogenesis may be alterations in genes of the homologous recombination (HR) system, which are involved in the repair of double-strand DNA breaks, cell cycle regulation, etc. [2]. Such alterations may manifest as dysfunction (accumulation of mutations, large chromosomal rearrangements, changes in expression, etc.) in key homologous recombination genes, particularly *BRCA1* and *BRCA2*. This dysfunction leads to the formation of a deficiency in the repair of double-strand DNA breaks, or homologous recombination deficiency (HRD) [3]. Cells with HRD cannot efficiently use the conservative homologous recombination mechanism. This deficiency leads to the repair of these DNA damages using non-conservative, potentially mutagenic mechanisms, such as non-homologous end joining and single-strand annealing, which can lead to genomic instability [4]. From an evolutionary standpoint, it is hypothesized that tumors benefit from developing a “mutator phenotype”, primarily driven by dysregulation of homologous recombination. The mechanisms of HRD formation therefore should increase due to the expansion of the frequency and spectrum of impairments. Initial mutations in HR genes can be compounded by deletions, methylation, downregulation of expression, etc., which can lead to the formation of more aggressive clones.

Chemicals that induce mutations and other genotoxic changes are particularly valuable for modeling carcinogenesis, as they directly initiate tumor development. Among these is 3-methylcholanthrene (MC), which causes DNA damage through metabolic activation by cytochrome P450 enzymes. The resulting reactive metabolites form covalent DNA adducts, leading to mutations. This leads to disruption of normal DNA reading and replication, which can promote genomic instability [5,6]. 3-methylcholanthrene is a polycyclic aromatic hydrocarbon (PAH) compound with pronounced carcinogenic properties, widely used in preclinical studies to mimic tumor formation processes in vivo and in vitro [7,8]. MC is a classic example of a procarcinogen; following metabolic activation in vivo, it is converted into reactive metabolites that bind to DNA, triggering a cascade of mutagenic events [9]. Trichloroacetic acid (TCA) can also be used to study carcinogenesis processes. It is another organic compound which is widely used as a model agent to induce cell proliferation and damage, particularly in the liver and kidneys, and has demonstrated toxicity and tumor-promoting properties [10]. Animal studies have shown that prolonged TCA exposure promotes hyperplasia, exacerbation of tissue damage, and induces epigenetic changes that increase the risk of malignant transformation [11].

To date, data on the gene expression profiles of tumors induced by these specific chemical carcinogens remain scarce. Existing evidence suggests a link between chemical exposure and homologous recombination (HR) dysfunction. For instance, exposure to PAHs at environmentally relevant doses was shown to dose-dependently reduce *BRCA1* expression in MCF-7 breast cancer cells [12]. Earlier studies showed that PAHs also suppress *BRCA1* in vitro and in vivo [13]. In rats, a single exposure to 3-methylcholanthrene caused prolonged induction of aryl hydrocarbon receptor (AhR)-regulated genes (e.g., *CYP1A1*) and genes involved in toxic response and DNA repair, associated with sustained inflammation and liver damage [14]. In a model of lung cell malignant transformation induced by 3-methylcholanthrene, specific genes were differentially expressed—*LCP1*, *PRSS37*, *BMP6*, *ZRANB3*, *MYB* and *OR1D5* were upregulated, while the *IQCA1*, *ZNF577*, *S100A7*, *IL1RN*, *VGLL1* and *CNN3* were downregulated [15]. Notably, these genes include genes involved in the regulation of the cell cycle, DNA repair, cell migration, etc. [16,17,18]. In this context, the use of mouse models has emerged as a powerful tool for studying the etiology and progression of cancer in vivo. However, investigating the effects of homologous recombination suppression in vivo remains challenging, particularly in the context of chemotherapy-induced carcinogenesis. For instance, mutations in *Rad51* have been shown to strongly predispose mice to lymphomas, while *Brca1* mutations promote the development of other tumor types [19]. Similarly, mouse models with partial loss of *Brca2* function exhibit increased susceptibility to carcinogenesis, with a predisposition to lymphomas [20]. In addition to the key HR genes *BRCA1* and *BRCA2* genes, other components of the HR pathway play important roles in both DNA repair and carcinogenesis. In particular, *Rad51* paralogs facilitate the recruitment of *Rad51* to DNA damage sites [21] and contribute to the formation and stabilization of the *Rad51* nucleoprotein filament. However, the precise role of each paralog has not yet been fully determined. Nevertheless, no *RAD51* mutation have been associated with cancer predisposition in humans, which constitutes the “RAD51 paradox” [22]. One potential explanation for this paradox is that mutations in mediator or accessory genes (such as *BRCA1* or *BRCA2*) prevent *RAD51* localization to damaged DNA, thereby promoting error-prone repair pathways [22]. Thus, studies in mouse models have shown that a decrease in *Rad51* activity in vivo does not promote tumor development, but rather confers protection against it. These data suggest that *Rad51*-mediated repair may facilitate tumor progression rather than function as a tumor suppressor [23,24].

Other low-penetrance HR genes, including *ATM*, *CHEK2*, *BRIP1*, and *BARD1*, have been extensively studied in the context of human breast carcinogenesis [25]. However, their function and contribution to tumor development in mouse models remain poorly characterized. Currently, data on the regulation of these genes in mouse models are limited, hindering a comprehensive understanding of their involvement in DNA repair and tumorigenesis [25].

Therefore, analyzing alterations in HR gene expression during carcinogenesis can enable us to determine how cells respond to genotoxic stress and how these processes can be disrupted in tumor tissues [26]. Understanding these dynamics can determine the sensitivity of tumor cells to DNA-damaging agents, which is important for personalized cancer treatment [26]. Furthermore, this study is important from the point of view of studying the mechanisms of the “pulsating” functionality of HR genes during tumor evolution. Consequently, the aim of this work was to evaluate the expression of key genes in the homologous recombination system in chemically induced carcinogenesis in mice.

## 2. Materials and Methods

### 2.1. Animals

A total of 40 outbred male ICR (CD-1) mice were used in this study (animals were provided by the Goldberg Research Institute of Pharmacology and Regenerative Medicine, Tomsk, Russia). Housing, care, and all manipulations with the animals were performed in accordance with the European Convention for the Protection of Vertebrate Animals Used for Experimental and Other Scientific Purposes (ETS N 123). The animals were kept under standard conditions at a temperature of 22 ± 2 °C and a relative humidity of 50–60%, with a 12 h light/dark cycle (from 8:00 AM to 8:00 PM). Food and water were provided ad libitum. Specialized feeds for laboratory animals from Delta Feeds (LBK 120 C-19, Novosibirsk, Russia) were used as the feed, with a metabolizable energy content of 2500 kcal/kg and a crude protein content of 19%. The feed composition included cereals, high-protein components (plant and animal proteins), vegetable oil, amino acids, organic acids, and a vitamin–mineral complex. All procedures with the animals were performed in the morning hours (from 9:00 AM to 11:00 AM local time) in accordance with the rules and recommendations for the humane treatment of animals used for experimental and other scientific purposes. Animal health and behavior were monitored daily by trained personnel, and any signs of discomfort or illness were promptly addressed by specialists.

### 2.2. Experimental Design

The study was designed following the 3R principles, reducing the number of animals to the necessary minimum and minimizing discomfort. Three groups of animals were formed for the experiment. Mice were randomly assigned to four groups (*n* = 10 per group) by weighing and randomization (according to the average weight, mean ± 10%). The mean body weight for the control groups was 27.2 ± 0.62 g; for study group #1—26.7 ± 0.44 g; group #2—26.6 ± 0.26 g. The following agents possessing direct or indirect genotoxicity were used as chemical agents for inducing oncogenesis: methylcholanthrene (10 mg, 3-Methylcholanthrene, 98%, Sigma-Aldrich #56-49-5, St. Louis, MO, USA) and trichloroacetate (250 g, Trichloroacetic acid, ≥99% Sigma-Aldrich #76-03-9, St. Louis, MO, USA).

### 2.3. Dose Selection

The dose of 3-methylcholanthrene and trichloroacetic acid were selected to limit the highest and lowest doses that cause tumors. Since MC is insoluble in water but readily soluble in organic solvents, the working solution for MC was prepared by dissolving 10 mg of MC (Sigma-Aldrich) in 300 µL of toluene, followed by the addition of 0.97 mL of olive oil. After this, 100 μL of the resulting solution was taken for administration. At this dilution, the dosage of the substance was approximately 40 mg/kg of animal weight, provided that 100 μL of solution was administered per 25 g of body weight. MC was administered subcutaneously in the withers of the laboratory animal in group #1 once. Control animals for the MC group received an equivalent volume of the olive oil/toluene vehicle alone.

It is worth noting that, according to published data, toluene can have a toxic effect on the behavioral characteristics of laboratory animals and some molecular parameters in the body [27]. However, cytochrome P4502A13 enables the effective metabolization of minimal doses of this substance in the body [28]. TCA was administered intraperitoneally at a dose of 250 μL per 25 g of body weight at a dosage of 25 mg/kg weekly for three months. Control animals for the TCA group received an equivalent volume of sterile saline [29]. Animal body weight was measured to track the overall condition of the organism. The euthanasia time was chosen based on the literature and empirical research data that established appropriate latency periods for tumor development at the selected doses. After subcutaneous administration of the drug, mice were euthanized after the appearance of tumors and their growth to a volume of at least 1 cm^3^. Tumor growth was assessed semi-quantitatively using a macroscopic scoring system as follows: 0 points—no visible macroscopically tumor; 1 point—limited tumor growth (diameter of tumor nodules 1–2 mm); 2 points—moderate tumor growth (diameter of tumor nodules 2–4 mm); 3 points—pronounced tumor growth (diameter of tumor nodules more than 4 mm).

### 2.4. Euthanasia

The procedure for humane euthanasia of animals was carried out after approximately 3 months using a CO_2_ asphyxiation with a gradual fill in gas concentration [30]. An autopsy of the animals to identify the tumor was carried out according to the method of autopsy and extraction of organs of laboratory animals outlined in Reference [31]. After removal, the tumor tissue was placed in RNAlater solution (Thermo Fisher Scientific Inc., Waltham, MA, USA). After 24 h incubation at +4 °C, tumor samples were stored at −80 °C for subsequent DNA and RNA extraction.

### 2.5. Morphological Study

To study the nature of morphological changes and confirm the presence of tumor tissue in the samples, suspected tumor tissue and potential metastases were fixed in 10% neutral-buffered formalin for 18–24 h. The material was then processed using standard methods, with embedding in paraffin. Serial sections with a thickness of 4–5 μm were prepared from paraffin blocks [32]. Microscopic slides were stained with hematoxylin and eosin solutions, prepared according to generally accepted protocols. Morphological examination was performed using an Axio Scope. A1 light microscope from Karl Zeiss, (Oberkochen, Baden-Württemberg, Germany). Microscopic evaluation was performed according to generally accepted criteria [33].

### 2.6. RNA Isolation

Total RNA was isolated from the cell suspension using the RNeasy Plus mini Kit (Qiagen, Hilden, Germany) according to the manufacturer’s instructions. RNA concentration was quantified using a Qubit 4.0 fluorometer (Thermo Fisher Scientific Inc., Waltham, MA, USA), yielding 100–250 ng/μL. RNA integrity was assessed via capillary electrophoresis on a TapeStation instrument (Agilent Technologies, Santa Clara, CA, USA) and an R6K ScreenTape kit. The RNA Integrity Number (RIN) ranged from 4.6 to 8.9.

### 2.7. Quantitative Real-Time qPCR

The expression levels of genes involved in homologous recombination—*Brca1*, *Brca2*, *Atm*, *Bard1*, *Brip1*, *Cdk12*, *Chek1*, *Chek2*, *Fancl*, *Palb2*, *Ppp2r2a*, *Rad51b*, *Rad51c*, *Rad51d*, *Rad54l*, and *Parp1*—were analyzed by reverse transcription quantitative PCR in real-time (RT-qPCR) using TaqMan technology on a Rotor-Gene-6000 amplifier (Qiagen, Hilden, Germany). PCR was performed in triplicate in a volume of 15 μL containing 250 μM dNTPs (Sibenzyme, Novosibirsk, Russia), 300 nM forward and reverse primers, 200 nM probe, 2.5 mM MgCl_2_, 1x SE buffer (67 mM Tris–HCl pH 8.8 at 25 °C, 16.6 mM (NH_4_)_2_SO_4_, 0.01% Tween-20), 2.5 units HotStart Taq polymerase (Sibenzyme, Novosibirsk, Russia) and 50 ng cDNA. The two-step amplification program included 1 cycle at 94 °C, with a 10 min pre-denaturation, followed by 40 cycles at 94 °C for 10 s and another 40 cycles at 60 °C for 20 s. Two reference genes, *Gapdh* (glyceraldehyde-3-phosphate dehydrogenase) and *Actb* (actin beta), were used as reference genes, and the expression level of the genes was normalized to these genes’ expression in the normal tissue and measured in arbitrary units. The relative gene expression was estimated using the Pfaffl method [5]. RNA isolated from normal tissue adjacent to the tumor was used as a calibrator.

### 2.8. Microarray Analysis

Transcriptome analysis was performed on the Clariom™ S Assay, mouse microarray platform (Thermo Fisher Scientific Inc., Waltham, MA, USA). RNA with a RIN ≥ 7.0 was used for the experiment. Sample preparation, hybridization, and scanning procedures were performed according to the manufacturer’s protocol on an Affymetrix GeneChip^®^ Scanner 3000 7G system (Affymetrix, Santa Clara, CA, USA). Microchip data analysis was performed using Transcriptome Analysis Console (TAC) software 4.0. The expression level for each sample was expressed as a base-two logarithm. Data normalization was carried out as described in Reference [34].

### 2.9. DNA Isolation

DNA was isolated from tumor cells using the QIAamp DNA mini Kit (Qiagen, Hilden, Germany) according to the manufacturer’s instructions. DNA concentration was measured using a Qubit 4.0 fluorometer (Thermo Fisher Scientific Inc., Waltham, MA, USA). The concentration ranged from 50 to 120 ng/μL. DNA integrity was assessed using capillary electrophoresis on a TapeStation instrument (Agilent Technologies, Santa Clara, CA, USA) using an Agilent Genomic DNA ScreenTape kit.

### 2.10. Digital PCR

Digital PCR (QIAcuity Digital PCR System (Qiagen, Hilden, Germany)) was used as a method for analyzing the copy number of the *Brca1* gene. Nanoplate 26K 24-well plates, with a coverage of 26,000 partitions, were used for the analysis. FAM and/or ROX were used as fluorescent dyes using TaqMan technology. The analysis of copy number variations by digital PCR included determining the number of targets and reference loci using a duplex method. In the QIAcuity Software Suite 2.0.20, the number of copies was determined by calculating the ratio of target molecules concentration to reference molecules and multiplying this ratio by the number of reference species copies in the genome (usually 2). The *Ap3b1* gene (adaptor-related protein complex 3 subunit beta 1), recommended by the manufacturer, was selected as the reference gene.

CNA = (A/B) × NB

A = concentration of target molecules (number of positive partitions of the *Brca1* gene);

B = concentration of reference molecules (number of positive partitions of the *Ap3b1* gene);

NB = number of copies of reference loci in the genome (usually 2).

Error bars for CN estimation in QIAcuity Software Suite are 95% of Poisson confidence intervals.

### 2.11. Statistical Analysis

Statistical data processing was performed using the application software package “Statistica 8.0” (StatSoft Inc., Stanford Research Park Palo Alto, CA, USA). The arithmetic mean and standard error were calculated for each sample. The Wilcoxon–Mann–Whitney test was used to test the hypothesis about the significance of differences between the studied groups.

## 3. Results

**Figure 1:**
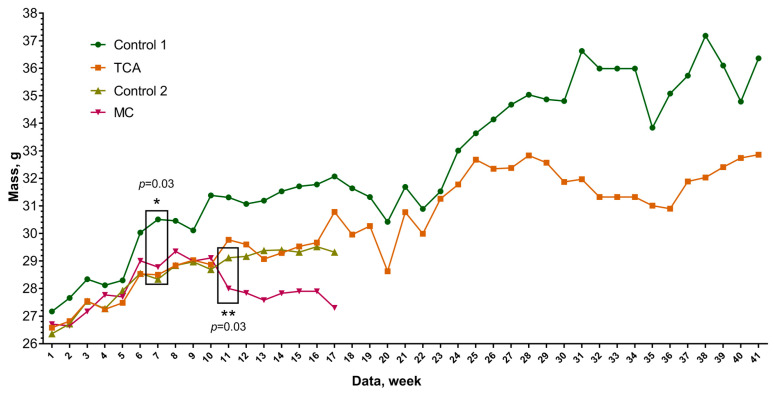
Dynamic weight changes in the study animal groups during the observation period. Note: MC—group of animals administered methylcholanthrene; TCA—group of animals administered trichloroacetate; * and **—statistically significant differences.

In the first stage of the study, body weight changes in all animal groups were assessed over the observation period of 30 December 2023–24 November 2024 (Figure 1).

Animals in the MC-treated group (#1) gained weight during the initial 1.5 months, while control animals maintained steady weight throughout. The Wilcoxon–Mann–Whitney test was used to test the hypothesis about the significance of weight differences between the studied groups. As a result of the analysis, it was found that a statistically significant change in the weight of the animals occurred (*p* = 0.03) and amounted to 29.1 ± 0.75 in the control groups versus 28.0 ± 0.62 in the study group (Figure 1) after 3.5 months of observation (Week 11), which corresponded to the approximate period of tumor formation. Then, a tendency to decrease in body weight was observed in this study group: Week 12—29.8 ± 1.07 vs. 27.8 ± 0.65 (*p* = 0.04); Week 13—29.4 ± 0.83 vs. 27.6 ± 0.48 (*p* = 0.004); Week 14—29.4 ± 0.84 vs. 27.8 ± 0.46 (*p* = 0.001); Week 15—29.3 ± 0.83 vs. 27.9 ± 0.46 (*p* = 0.001); Week 16—29.5 ± 0.85 vs. 27.9 ± 0.51 (*p* = 0.001); Week 17—29.3 ± 0.52 vs. 27.2 ± 0.44 (*p* = 0.0003).

### 3.1. Morphological Study of Tumors Induced by MC

**Figure 2:**
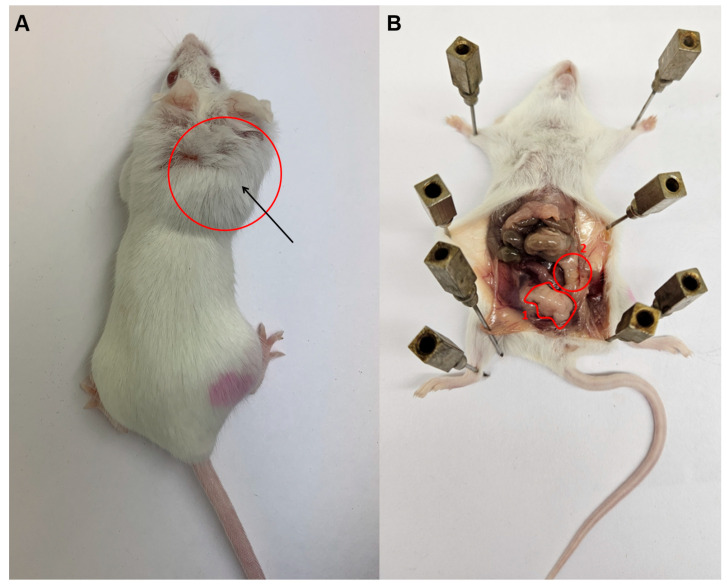
External manifestation of methylcholanthrene’s action at the injection site (after 2 months of observation) (**A**) and the boundaries of intraperitoneal tumors in a laboratory animal after administration of trichloroacetic acid (**B**). Note: 1—adipose tissue fragments; 2—adipose tissue fragments containing solid nests of highly polymorphic tumor cells (description in text).

Subcutaneous administration of 3-methylcholanthrene induced tumor formation at the injection site in all mice in the group within approximately two months (Figure 2A). After euthanasia, the animals were autopsied, followed by pathological and histological examination for the presence of malignant cells.

**Figure 3:**
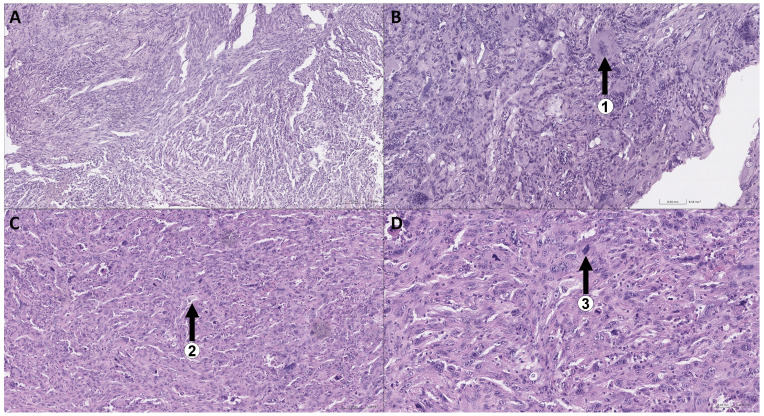
Microscopic preparations of tumor samples No. 6 and No. 5 at 10× magnification (**A**,**C**) and 20× magnification (**B**,**D**), respectively.

As a result of the analysis, within the preparations, the morphological picture is that of a pleomorphic cell tumor, forming differently sized, multidirectional, fascicular-swirling structures and large solid foci composed of markedly polymorphic spindle-shaped and epithelioid cells (Figure 3А). Against this background, multiple giant multinucleated cells (1) and monster cells are found (2). The tumor stroma is represented by thin strands of mature fibrous connective tissue with uneven, moderately pronounced lymphoplasmacytic infiltration. In some foci, large cells with clarified cytoplasm and large, round vesicular nuclei are found among the tumor cells (3). The tumors displayed high mitotic activity.

### 3.2. Morphological Study of Tumors Induced by TCA

TCA administration induced a statistically significant change in animal body weight as early as one month later (Week 7) (Figure 1). The average weight of the animals in the control group at this time was 30.5 ± 0.84 compared to the TCA group: 28.5 ± 0.29 (*p* = 0.03). The differences became stronger, with *p* = 0.01 for the group with TCA (28.9 ± 0.31) and the weight in the control group 31.4 ± 1.07 after three weeks (Week 10). The further trend of weight difference continues. At Week 17, an increase in the weight of the control groups to 32.1 ± 0.91 g is observed. In the group with TCA, the weight of the animals was 30.8 ± 0.49. The differences are statistically significant: *p* = 0.004. In the period from the 17th week, small intra-abdominal formations were detected during palpation of the animals. Further examination of the animals showed that in the group with TCA administration, 50% (5/10) of animals showed the appearance of neoplasms (Figure 2B).

**Figure 4:**
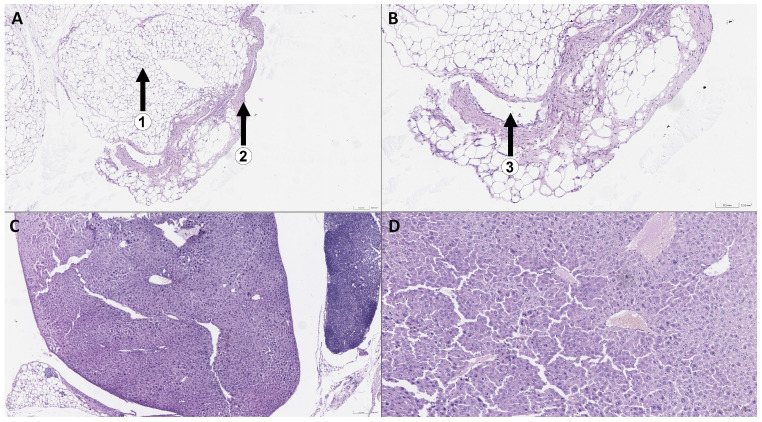
Microscopic preparations of neoplasm sample No. 6 at 5× (**A**) and 10× (**B**) magnification, as well as a fragment of liver tissue sample No. 1 at 5× (**C**) and 10× (**D**) magnification.

Figure 2B shows a gross image of an animal. The number 1 indicates the boundaries of the first neoplasm. Morphological examination showed that the preparation is represented by fragments of adipose tissue (1) with fibrous cords (2) and an abundance of thin-walled vessels (3). No reliable signs of the presence of tumor elements were found within the preparation (Figure 4A,B). This phenomenon is observed in all animals. In addition, the liver is enlarged in the entire group of mice with TCA administration compared to the control group. However, morphological analysis did not show the presence of tumor cells in the organ. In the preparation, there is a fragment of liver tissue of a typical histological structure. Hepatocytes are medium-sized, with a slightly granular, dense cytoplasm and round normochromic nuclei. The portal tracts are not structurally dilated. Structures of a large bile duct of normal structure are determined along the edge of the fragment (Figure 4C,D).

**Figure 5:**
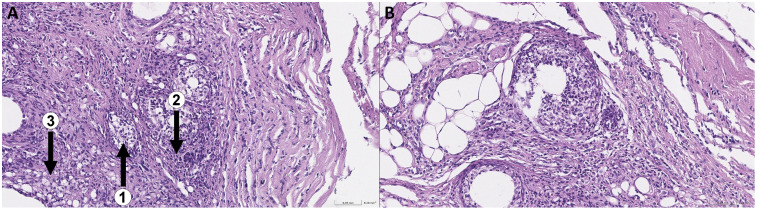
Microscopic preparations of tumor samples No. 3 (**A**) and No. 6 (**B**) at 20× magnification.

Under number 2 in Figure 2B, the boundaries of the second neoplasm are depicted. This tumor is represented by fragments of adipose tissue, interspersed with fragments of fibrous connective tissue of varying degrees of maturity. In some fields of view, small solid nests of markedly polymorphic small and medium-sized tumor cells can be identified, with faintly expressed pale cytoplasm and round hyperchromatic nuclei (1). The tumor cells are surrounded by uneven lymphoid infiltration with an admixture of plasmacytes, macrophages, and single eosinophils (2). Throughout the fragments, accumulations of foamy macrophages (3) and foci of hemorrhages are able to be identified (Figure 5).

### 3.3. Analysis of the Expression Level of Homologous Recombination Genes in Tumor Tissue

**Figure 6:**
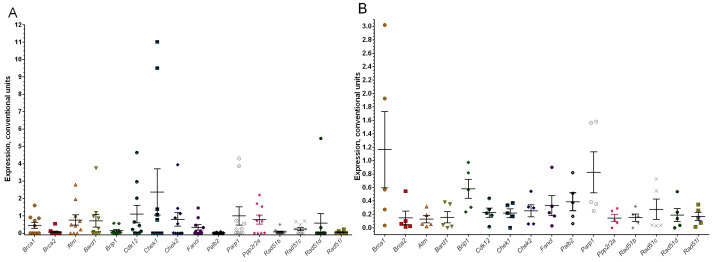
Expression level of the main genes of homologous recombination in a group of mice with the introduction of the carcinogen methylcholanthrene (**A**) and trichloroacetic acid (**B**). Note: The figure shows the mean expression levels of HR genes in arbitrary units, presented as mean ± standard error (SE).

The next stage of the study was to examine the parameters of the *Brca1* gene and other homologous recombination genes. In particular, the expression of the studied genes was determined in the obtained tumor samples. First of all, the expression of the *Brca1* and *Brca2* genes was evaluated (Figure 6). The average expression value in the first experimental group for the *Brca1* gene was 0.45 ± 0.17 and for *Brca2* this was 0.07 ± 0.05 (Figure 6A). For the second group, the *Brca1* expression value is higher, which is associated with the dropout of two samples beyond the normal expression range (greater than 1) and amounted to 1.16 ± 0.56, and 0.14 ± 0.1, respectively, for *Brca2* (Figure 6B). It should be noted that the analysis of *Brca1* copy number in these samples showed a normal copy number and amplification (Supplementary Materials, Table S1).

Analysis of the expression of other repair genes showed that a low or absent expression level is predominantly observed (Figure 6). At the same time, in the group of animals treated with methylcholanthrene, overexpression was established only for two genes: *Cdk12* (1.1 ± 0.5) and *Chek1* (2.37 ± 1.32) (Figure 6A). Under the action of trichloroacetic acid, overexpression was shown for *Brca1* and *Parp1* in the resulting tumors (Figure 6B). Interestingly, correlation analysis of the gene expression methylcholanthrene-treated group revealed significant positive correlations between *Cdk12* expression and several DNA repair genes, including *Brca1* (r = 0.68), *Atm* (r = 0.71), *Bard1* (r = 0.84), *Fancl* (r = 0.74), *Palb2* (r = 0.89) and *Parp1* (r = 0.88), *p* < 0.05. These findings further support the regulatory role of *Cdk12* [35]. When analyzing the presence of DNA copy number aberrations in the studied samples, it was found that the frequency of deletions in the *Brca1* gene in group #1 is 60%, and 40% of animals have a normal gene copy number (Supplementary Materials, Table S2). In group 2 (TCA-treated), among five animals, two exhibited a gene deletion, one had a normal copy number, and two experienced gene amplification, which was consistent with the expression level of this gene (Figure 6B). Nevertheless, it is currently difficult to draw conclusions regarding a correlation between the presence of aberrations and gene expression levels. This may be attributed to individual variability among the studied samples.

### 3.4. Microarray Analysis of HR Genes Expression

Next, a microarray analysis of the expression of homologous recombination system genes was performed (Table 1). The evaluation of expression was carried out by means of the averaged expression value for all the studied tumor tissue samples and was similarly compared with the expression in normal tissue.

Among the studied genes, 14 were hypoexpressed in the tumor compared to normal tissue (Table 1) at *p* < 0.05. It is worth noting that a high level of difference is characteristic of the *Rad50*, *Rad51*, *Brca1*, *Brca2* and *Pold4* genes. The two genes *Rad52* and *Bard1* have an increased level of expression. A similar analysis was carried out for the second group of animals. It was found that a statistically significantly low level of expression in the tumor is characteristic of the *Rad50, Mre11a*, *Rpa1*, *Rad51*, *Brca2*, *Pold1*, *Pold3*, *Pold4* genes, as well as *Brca1*, *Parp1*, *Palb2*, *Brip1*, *Bard1*. The genes *Atm*, *Nbn*, *Rad54b* have almost equal expression values to normal tissue (Table 1). All differences are statistically significant at *p* < 0.05.

In the final stage of the study, a microarray analysis of the expression of homologous recombination system genes was performed in the group of laboratory animals depending on the size of the tumor nodule (Supplementary Materials, Table S2). The evaluation of expression was carried out for tumor samples in the group of animals with methylcholanthrene administration. For this group, it was possible to collect material at different stages of tumor development (from the moment of visual appearance of the tumor nodule to maximum development). Thus, it was possible to study changes in the expression of homologous recombination genes at different time intervals.

The material was taken from the animals in the following order: animals 4, 5, 8, then 7, 9, 10, 1, 2, 3, and finally animal 6. The interval between euthanasia and material collection was approximately 1 week. As a result of the microarray analysis, the following data were obtained: with a small size of the tumor nodule in mice 4, 5, and 8, an increase in the expression of repair genes is predominantly observed, or the expression level does not change relative to normal tissue. In particular, for mouse 4, there was a statistically significantly high level of expression for the *Pold2, Pold4* and *Brip1* genes. For mouse 5, no changes were established, while for mouse 8, hyperexpression was shown for the *Atm, Rad50*, *Nbn*, *Mre11a*, *Rpa1*, *Brca1*, *Pold1*, *Pold2*, *Pold3*, *Pold4*, *Parp1*, *Palb2*, *Brip1*, and *Bard1* genes, and a decrease in expression was established for three genes (*Rad51, Brca1*, *Rad54b*) (Supplementary Materials, Table S2). For mouse 7, 13 out of 18 genes have a low level of expression in tumor tissue. For mouse 9, no noticeable changes in the expression of HR genes were established. It is worth noting that for mice 10, 1, 3, and 6, as the tumor nodule increases, the frequency of homologous recombination genes with hypoexpression increases.

## 4. Discussion

MC and TCA are widely used in toxicological and oncological research to investigate the mechanisms of carcinogenesis. The present study demonstrates that the use of these carcinogens in animal models enables a deeper understanding of their biological effects, toxicity profiles, and the molecular and genetic characteristics of the tumors they induce, which cannot always be fully reproduced in in vitro models. This study demonstrated that the administration of 3-methylcholanthrene to animals caused tumor formation, which confirms its carcinogenic properties. The dose of MC was chosen based on studies that showed that the introduction of 6–10 mg of MC is necessary to induce kidney tumors in rats. Thus, preliminary exposure to MC (introduction into the kidney in a small dose, insufficient during the study period to induce kidney tumors) with subsequent applications of this substance to the skin led to an increased blastomogenic effect, which was expressed both in the early appearance of precancerous changes and in the more frequent development of tumors at the site of primary MC administration (in the kidney) [36].

Homologous recombination, which is responsible for the repair of DNA double-strand breaks, plays an important role in maintaining genomic stability and preventing carcinogenesis [37]. However, when exposed to carcinogens, such as 3-methylcholanthrene, the HR system may experience a deficiency in activity, leading to the accumulation of mutations and, as a consequence, tumor formation [38]. An interesting finding emerged from the analysis of *Cdk12* and *Brca1* gene expression in tumor tissue. A significant positive correlation was observed between *Cdk12* expression and the genes *Brca1*, *Atm*, *Bard1*, *Fancl*, *Palb2*, and *Parp1*. At the same time, according to the literature data, impairment of *Cdk12* functional activity leads to defects in DNA repair, resulting in genomic instability and decreased expression of certain homologous recombination genes, such as *Brca1, Fanci*, and *Fancd2* [35,39]. Particular attention was paid to the *Brca1* gene, the expression of which was significantly reduced in tumor tissues. Cases have been described in the literature where mutant mice with *Brca1* defects (Brca1tr/tr) developed various types of tumors, including breast carcinomas and lymphomas without additional genetic changes, such as inactivation of the *Trp53* gene. Our data support these results and confirm the importance of disruptions in *Brca1* function in the carcinogenesis mechanism [40]. In addition, an assessment of the copy number of the *Brca1* gene was carried out using digital PCR (Supplementary Materials, Table S1). In the group of animals exposed to MC, a high frequency of deletions in the *Brca1* gene was revealed, which correlates with the low expression of this gene in tumor tissues and confirms the assumption about its role in maintaining genomic stability. In a study by Rhon-Calderón E. A. et al., the authors showed that exposure to MC altered the epigenomic landscape primarily in the promoter regions of genes associated with xenobiotic biotransformation (*Cypa1a*), Notch signaling (*Jag1*), stress and tumor progression (*Dnajb*), apoptosis (*Bax* and *Caspase-9*), cell survival (*Cdk2*) and expression of growth factors (*Igf2*), transcription factor (*Sp1*) and adhesion molecules (*Adamts1*, *Icam1*) [41]. These findings highlight the importance of considering not only the expression profile of the tumor in the present study but also additional factors such as methylation of HR genes, DNA copy number aberrations, and mutations, all of which may affect the activity of key homologous recombination genes. An integrated approach is therefore likely to provide will a more comprehensive and accurate assessment of the spectrum of alterations and the regulation of DNA repair processes.

Analysis of the expression of homologous recombination (HR) system genes revealed significant changes depending on the size of the tumor nodule (Supplementary Materials, Table S2). It was shown that with small tumor sizes, the expression of repair genes either increased or did not change compared to normal tissues, which may be associated with the activation of compensatory mechanisms in the early stages of tumor growth. As the tumor nodule increased, a progressive decrease in the expression of key genes was observed both in the excision repair system (*Ddb2* and *Pole*) and in the HR system (9 genes (*Atm*, *Rad50*, *Topbp1*, *Brca1*, *Brip1*, *Palb2*, *Rad52*, *Brca2*, *Sycp3*)), which indicates depletion of the repair potential as the tumor develops. Although the data obtained on clinical material showed that various changes in gene expression are associated with varying degrees of tumor malignancy, breast tumors of grades I and III malignancy demonstrate reciprocal patterns of gene expression, while tumors of grade II malignancy demonstrate a hybrid pattern of signatures of grades I and III malignancy [42]. It was further shown that as tumors of various localizations progress, not only does a change in the expression profile occur, but there is also an accumulation of chromosomal instability and a change in epigenetic mechanisms, which contributes to the appearance of more aggressive forms of tumors [43]. This is consistent with our hypothesis and the data obtained on changes in the expression profile of HR genes.

Our study revealed overexpression of the *Rad52* and *Bard1* genes (Figure 6). It can be assumed that increased expression of these genes in tumors may represent a compensatory mechanism in the context of *Brca1/2* dysfunction [44]. A number of studies support this hypothesis [45,46]. *Rad52* expression can trigger or regulate the initiation of repair processes, thereby influencing the activity and functionality of the entire pathway. In response to DNA damage arising from both environmental factors and endogenous metabolic processes, *Rad52* may enhance repair capacity through excision repair and homologous recombination, contributing to the maintenance of genome stability [47]. As for the *Bard1*, Shakya R. et al. demonstrated that *Bard1* inactivation induces basal-like breast carcinomas with a frequency, latency, and histopathological characteristics indistinguishable from those observed in mice carrying a *Brca1* mutation or a *Bard1/Brca1* double mutation [48]. These findings indicate that *Bard1* functions as a key tumor suppressor gene, together with *Brca1*, and that *Brca1*-mediated tumor suppression is largely dependent on the *Bard1/Brca1* heterodimer. However, the question remains as to why high *Bard1* expression is observed despite reduced *Brca1* expression. Interestingly, in our previous in vitro study, it was found that under continuous exposure to cytostatic agents, cell lines with *BRCA1* dysfunction acquired genetic alterations characterized by amplifications of HR genes (including *BARD1*) and increased expression levels [49].

There are no studies regarding the effect of trichloroacetic acid on the expression of HR genes, either in normal or tumor tissue (Figure 6, Table 1). However, it has been shown that in the livers of mice exposed to TCA, a decrease in methylation was found in the promoter regions of the c-Jun and c-Myc genes alongside an increase in the levels of their mRNA and proteins [50]. In addition, it has been shown that trichloroacetic acid has a carcinogenic effect on the liver of B6C3F1 mice [51] and promotes the development of liver tumors. Moreover, a high level of hypermethylated DNA is recorded in these tumors [52].

## 5. Conclusions

Thus, the present study demonstrates that 3-methylcholanthrene and trichloroacetic acid induce structural alterations in key homologous recombination genes, including reduced expression of the *Brca1/2* genes and a high frequency of deletions, which may contribute to the carcinogenic effects of these compounds. In addition to DNA repair pathways, significant changes were observed in the expression profiles of genes involved in xenobiotic biotransformation, apoptosis, and cell proliferation. These findings underscore the importance of a comprehensive analysis of aberrant HR gene states for understanding the mechanisms of carcinogenesis and highlight potential avenues for the development of novel diagnostic and therapeutic strategy in oncology.

### Study Limitations

This study has several limitations that should be considered when interpreting the results. To confirm the accumulation of DNA damage in tumor cells following suppression of homologous recombination genes, additional validation using immunohistochemistry or Western blot analysis is required, as mRNA expression levels are variable and do not always reflect protein activity. At this stage of our research, these experiments were not feasible due to the specific characteristics and the need to optimize staining conditions for the mouse model. Nevertheless, we acknowledge the importance of this data and plan to include them in future studies to further validate our findings.

## Figures and Tables

**Table 1:** The expression level of homologous recombination genes that differ statistically significantly in normal and tumor tissue in the study groups.

Genes	Group No. 1 with the Introduction of Methylcholanthrene			Group No. 2 with the Introduction of TCA		
	Normal Tissue Avg (Log2)	Tumor Tissue Avg (Log2)	Fold Change	Normal Tissue Avg (Log2)	Tumor Tissue Avg (Log2)	Fold Change
*Atm*	9.03	6.93	4.3	8.86	7.7	2.24
*Rad50*	11.6	8.39	9.22	11.26	8.64	6.14
*Nbn*	9.23	7.58	3.14	9.2	8	2.29
*Mre11a*	10.04	8.00	4.12	9.94	8.16	3.44
*Rpa1*	13.24	10.59	6.28	13.82	10.64	9.09
*Rad51*	8.72	4.32	21.21	8.48	4.51	15.6
*Brca1*	10.05	4.12	61.07	10.31	4.62	51.67
*Brca2*	10.85	6.7	17.86	10.96	6.23	26.57
*Rad52*	2.92	3.06	−1.1	3.16	3.13	1.02
*Rad54b*	5.78	4.99	1.73	5.71	4.55	2.23
*Pold1*	7.44	4.54	7.49	7.11	4.42	6.42
*Pold2*	10.43	9.86	1.49	10.51	9.69	1.76
*Pold3*	11.96	9.44	5.74	11.9	10.03	3.64
*Pold4*	9.19	5.84	10.18	9.29	5.8	11.24
*Parp1*	10.21	7.85	5.11	10.26	8.22	4.11
*Palb2*	6.55	4.67	3.68	7.41	4.12	9.81
*Brip1*	9.69	8.33	2.57	9.92	7.87	4.14
*Bard1*	5.12	7.08	−3.9	7.53	4.29	9.46

Note: Fold change is an indicator of how many times the expression level changes.

## Data Availability

The original contributions presented in this study are included in the article/Supplementary Materials. Further inquiries can be directed to the corresponding author.

## Supplementary Materials

The following supporting information can be downloaded at https://www.mdpi.com/article/10.3390/cimb48030275/s1.

## Abbreviations

The following abbreviations are used in this manuscript:

MC	3-methylcholanthrene
TCA	trichloroacetic acid
PAH	polycyclic aromatic hydrocarbon
