# Supplementary material for: Assessment of Homologous Recombination System Gene Expression in Chemologically Induced Carcinogenesis In Vivo Models
Source: Curr Issues Mol Biol. 2026 Mar 4;48(3):275. doi: 10.3390/cimb48030275 (PMC13025133; doi:10.3390/cimb48030275)
Supplement: Supplementary file 1 [file cimb-48-00275-s001.zip › cimb-4154340-supplementary.pdf]

Table S1 Results of analysis of the presence of DNA copy number aberrations in the *Brca1* gene using digital PCR.

Group	№	*Brca1* (GREEN (FAM))			*Ap3b1* (ORANGE (ROX))			(Positive *Brca1*/ Positive *Ap3b1*) x2	CNA
		Total number of cells	Positive cells	Negative cells	Total number of cells	Positive cells	Negative cells		
Group No. 1 with the introduction of methylcholanthrene	1	25253	11689	13564	25253	24892	361	0,9391	Loss
	2	25329	10915	14414	25329	23360	1969	0,9345	Loss
	3	24909	12289	12620	24909	24363	546	1,0088	Loss
	4	25461	25099	361	25461	25042	418	2,0045	n
	5	25461	25303	6484	25461	25287	175	2,0012	n
	6	25143	10172	14971	25143	22930	2213	0,8872	Loss
	7	24953	12805	12148	24953	24061	892	1,0643	Loss
	8	24869	24717	152	25005	24981	24	1,9788	n
	9	25467	18538	6929	25467	18387	7080	2,0164	n
	10	25004	11978	13026	25004	24096	908	0,994	Loss
Group No. 2 with the introduction of TCA	1	25066	10701	14365	25066	22944	2122	0,9327	Loss
	2	25123	9187	15936	25123	22587	2536	0,8134	Loss
	3	25425	18296	6167	25425	11601	13824	3,1541	Gain
	4	24811	10074	14737	24811	21820	2991	0,9233	n
	6	25435	19222	6213	25356	11874	13482	3,2377	Gain

Note: CNA - is a DNA copy number aberration, or DNA copy number anomaly; Loss is a deletion; n is the normal copy number of a gene; Gain is amplification.

Table S2 Results of microarray expression analysis of homologous recombination genes in mice of the first group (methylcholanthrene).

Genes	4 mouse		5 mouse		8 mouse		7 mouse		9 mouse		10 mouse		1 mouse		2 mouse		3 mouse		6 mouse	
	N tissue	T tissue	N tissue	T tissue	N tissue	T tissue	N tissue	T tissue	N tissue	T tissue	N tissue	T tissue	N tissue	T tissue	N tissue	T tissue	N tissue	T tissue	N tissue	T tissue
Atm	7.7	8.2	7.59	7.71	6.44	9.21	8.48	8.03	7.82	7.35	10.9	6.86	13.56	12.24	11.28	10.42	13.1	10.53	10.03	8.2
	-1.42*		-1.08		-2.84		1.37		1.38		16.44		2.49		1.81		5.94		1.78	
Rad50	8.64	8.58	8.6	8.28	4.47	11.47	11.76	9.63	8.86	8.94	9.79	5.54	10.62	7.56	5.53	5.08	13.08	6.19	10.6	8.58
	1.04		1.24		-9.08		4.38		-1.05		19.03		8.33		1.36		118.61		8.08	
Nbn	8	8.51	7.66	7.69	5.51	9.2	8.96	8.26	8.65	8.83	3.36	4.68	7.54	4.62	12.78	7.1	9.24	11.59	8.23	8.51
	-1.42		-1.02		-2.85		1.63		-1.14		-2.51		7.52		51.13		-5.07		1.64	
Mre11a	8.16	7.83	8.12	7.88	9.08	10.44	10.58	7.61	7.67	7.96	7.45	4.43	11.79	10.68	4	3.91	4.71	6.16	10.54	7.83
	1.26		1.18		-5.89		7.8		-1.22		8.1		2.16		1.07		-2.73		4.64	
Rpa1	10.64	11.06	10.86	10.41	3.6	13.23	13.01	11.26	10.57	11.06	12.4	10.19	8.73	6.73	4.83	4.8	6.98	8.63	12.24	11.06
	-1.34		1.37		-7.06		3.35		-1.4		4.62		4.02		1.02		-3.14		4.53	
Rad51	4.51	4.7	5.18	4.36	9.24	8.69	8.21	3.96	4.35	3.97	8.79	6.12	14.09	11.44	4.39	5.63	11.15	8.53	9.72	4.7
	-1.14		1.77		2.11		19.08		1.3		6.35		6.24		-2.36		6.13		16.28	
Brca1	4.62	4.82	3.91	4.55	9.42	10.59	10.33	5.02	5.63	4.61	11.23	9.7	8.54	5.93	8.04	7.53	10.87	7.31	9.05	4.82
	-1.15		-1.56		-6.1		39.69		2.02		2.88		6.11		1.42		11.76		37.5	
Brca2	6.23	6.15	6.65	6.24	11.47	10.78	10.87	7.24	6.2	6.57	10.92	9.33	6.42	7.81	3.29	4.27	11.62	7.73	9.81	6.15
	1.06		1.32		2.15		12.42		-1.29		3.01		-2.64		-1.98		14.79		26.08	
Rad52	3.13	2.98	3.4	3.27	6.76	3.15	3.26	2.91	3.29	2.74	15.54	11.7	7.53	4.57	9.96	5.71	8.6	6.7	2.9	2.98
	1.11		1.09		1.09		1.28		1.46		14.35		7.76		19.06		3.74		-1.04	
Rad54b	4.55	4.68	4.4	4.33	7.58	5.93	5.14	4.71	4.83	4.71	4.02	8.05	9.11	6.51	10.14	3.61	13.13	9.39	6.68	4.68
	-1.1		1.05		3.02		1.35		1.09		-16.27		6.09		92.24		13.3		2.14	
Pold1	4.42	5.29	4.54	4.53	6.78	7.28	7.21	4.97	5.55	4.22	3.59	4.65	10.17	8.19	14	12.5	8.75	7.05	6.94	5.29
	-1.82		1.01		-6.74		4.7		2.52		-2.08		3.95		2.82		3.26		4.44	
Pold2	9.69	10.72	9.5	9.35	8.86	10.56	10.64	10.59	10.61	10.55	9.78	3.86	7.81	9.08	9.06	8.01	9.28	10.92	10.44	10.72
	-2.03		1.11		-2.31		1.04		1.04		6.22		-2.41		2.08		-3.14		-1.22	
Pold3	10.03	9.37	9.68	9.4	8.53	12.07	11.94	10.1	10.16	9.15	8.35	11.1	11.18	12.5	15.1	11.73	7.96	8.98	12.06	9.37
	1.59		1.22		-6.37		3.59		2.02		-6.72		-2.49		10.35		-2.03		6.05	
Pold4	5.8	7.39	6.01	6.22	3.92	9.24	8.87	7.14	6.74	6.37	8.26	5.55	6.23	4.46	4.08	4.26	5.32	2.99	10	7.39
	-3.01		-1.15		-8.17		3.33		1.29		6.56		3.39		-1.13		5.02		3.47	
Parp1	8.22	8.01	8.1	7.92	7.56	10.43	10.06	8.74	8.51	8.14	3.91	5.15	14.11	15.33	7.53	7.49	12.91	9.3	10.24	8.01
	1.15		1.14		-5.71		2.5		1.29		-2.36		-2.33		1.03		12.26		4.57	
Palb2	4.12	4.89	4.86	4.8	5.06	7.1	6.72	4.65	4.52	4.06	10.72	11.99	14.46	10.63	8.1	13.47	5.09	8.39	6.99	4.89
	-1.71		1.04		-4.92		4.21		1.38		-2.41		14.24		-41.43		-9.85		3.15	
Brip1	7.87	8.93	8.39	8.1	5.66	10.06	10.23	8.69	8.43	8.54	4.04	6.37	9.18	5.12	8.07	5.8	11.05	8.29	9.74	8.93
	-2.08		1.22		-3.89		2.91		-1.08		-5.02		16.69		4.83		6.81		1.7	
Bard1	4.29	4.99	5.25	4.81	3.04	7.57	7.53	4.93	4.96	4.57	8.43	6.87	8.39	5.09	7.74	8.78	6.05	3.39	8.24	4.99

	-1.63	1.36	-6.76	6.06	1.31	2.96	9.84	-2.06	6.29	4.25

*Note: * - Fold Change; Green - increased expression; red - decreased expression. Highlighted data show statistically significant differences (at p<0.05)*
